# Relationship of Coping Strategies and Quality of Life: Parallel and Serial Mediating Role of Resilience and Social Participation among Older Adults in Western Philippines

**DOI:** 10.3390/ijerph181910006

**Published:** 2021-09-23

**Authors:** Madonna S. Palmes, Sheilla M. Trajera, Gregory S. Ching

**Affiliations:** 1 College of Nursing, West Visayas State University, Iloilo City 5000, Philippines; madonna.palmes@wvsu.edu.ph; 2 Center for Linkages and International Affairs, Faculty, BSN MN and PhD Programs in Nursing, University of St. La Salle, Bacolod City 6100, Philippines; s.trajera@usls.edu.ph; 3 Graduate Institute of Educational Leadership and Development, Research and Development Center for Physical Education Health and Information Technology, Fu Jen Catholic University, New Taipei City 24205, Taiwan

**Keywords:** quality of life, older adults, resilience, social participation, coping strategies, Western Visayas, Philippines

## Abstract

Growing old is frequently linked with various challenges. Hence, it is important to understand how to age successfully. Drawing on the concept that an individual’s quality of life (QOL) is influenced by their demographics, coping strategies, resilience, and social participation, the current study reports on the findings of these variables among older adults in the Western Philippines. A total of 392 volunteer older adults were surveyed. Aside from the demographics such as age, gender, marital status, average monthly income, educational attainment, and health status, the data collected also included the 30-item coping strategies for the elderly, 14-item resilience scale, 12-item social participation scale, and 35-item older people’s QOL scale. Structural equation modeling was used to verify the parallel and serial mediating role of resilience and social participation within the relationship between coping strategies and QOL. Findings show that coping strategies alone are not enough to improve QOL. The only way to improve QOL is through resiliency, as well as the ability to participate in social activities. In essence, the QOL of older adults can be improved by encouraging them to have more social participation, and at the same time, understand how it takes to become resilient.

## 1. Introduction

Globally, the population is aging rapidly [1]. The aging population has now become a universal phenomenon. There are an estimated eight million senior citizens or older adults in the Philippines in 2018, a rise of around 3% from 2015 [2]. In the Western Philippines, the 2015 census shows there are around 7.5 million residents, and 472,000 of those are older adults aged 65 and above [3]. Being one of the most popular areas in the Philippines, the region offers excellent agricultural and fishing opportunities; more importantly, however, are the dazzling beaches that attract tourists from around the world [4]. Before the pandemic, the region experienced economic growth of 7.6% [5]. Despite this, 18.2% of older adults still live in poverty [6]. To alleviate the problem, various social protection programs are in place, including discounts on health care and pensions [7,8]. Although these services are available, access and awareness remain the major barriers to healthy aging [8]; therefore, older adults in Western Philippines can be considered as a case of interest.

In recent years, studies on the issue of aging have been gaining much attention [9,10,11,12]. Among the most important aspects of aging is to understand how to age without sacrificing quality of life (QOL) [13]. Quality of life as defined by the World Health Organization encompasses the complete physical, mental, and social well-being of an individual [14]. This also includes the capacity for physical and mental function and the ability to engage in normative social interactions [15]. Noting that the physical, psychological, emotional, and social capacities of an individual are highly influenced by their age, QOL, therefore, carries a general sense of well-being containing both aspects of happiness and satisfaction of life itself [16]. In other words, although aging is associated with vulnerabilities, many older adults still lead full and productive lives due to the quality of their lives [17]. Researchers have noted several distinct variables associated with QOL, such as certain demographics, coping strategies, resilience, and participation in social activities [18,19,20].

Certain demographics have been found to be related to QOL. In a study of one thousand elderly adults in South Africa, wherein demographics, perceived health, life satisfaction, and social support are analyzed, Galiana et al. [21] pointed out that those perceived as having the worst QOL and having the lowest life satisfaction and social support are believed to be the oldest people. Similarly, an analysis of 630 women in Europe also revealed that QOL is highly influenced by both education and age [22]. Likewise, gender and marital status also matter [21,23,24]. For instance, women are found to have poorer perceptions of their health than men, yet they have better social relationships and QOL. According to the same study, the average QOL of widows and widowers is lower than that of married older persons [21]. Additionally, a study in the Philippines has found that an individual’s financial capabilities directly affect their QOL; for example, poorer people are perceived to have lower QOL than more affluent ones [9]. Furthermore, education is hypothesized to lead to improvements in QOL, such as better health, life satisfaction, and social relations [24]. Therefore, younger people not living alone and those with a higher level of education appear to have a better QOL.

Understanding how older individuals cope with daily activities and social roles is another important aspect of aging [25,26]. Proactive approaches to aging are used to help promote the development of constructive, problem-orientated, and positive coping strategies [27,28]. Thus, coping strategies can be viewed as a positive approach to normal aging [29]. It has been suggested that positive coping strategies can improve QOL [30,31]. It is also true that, if used properly, positive coping strategies can improve QOL even in stressful situations [32,33]. According to these studies, QOL can be improved by an appreciation of humor, an adequate level of education (information), and a desire for help. In addition, Atal and Cheng’s [30] study showed that participants with higher coping tendencies had similar levels of QOL regardless of their financial status. According to their study, coping strategies played an important role in enhancing QOL than an individual’s socioeconomic status (SES). This finding is quite important for older adults in the Western Philippines, where around 12% of families live below the poverty line [34].

The resilience of a person also has an important effect on their QOL. Resilience is the ability to handle difficulties, stresses, and adversity well [35]. In a way, it can be seen as bouncing back from a difficult experience [36]. Importantly, it consists of the qualities that enable one to thrive despite adversity [37]. For the past two decades, scholars have examined the role of resilience in promoting QOL [38]. Numerous studies have demonstrated resilience as an important mediator of QOL [39,40,41]. Specifically, studies on older adults in the Philippines have noted the positive associations of resilience and coping strategies towards QOL [10,42]. Similar findings were also noted in a group of 119 middle-aged women in South Korea, wherein results showed that the QOL increases in proportion to one’s ability to cope and their level of resilience [43]. In essence, even if older individuals are healthy as they age, there is always a possibility that they will experience some period of functional decline, which could impact their QOL [44]. Resilience is, therefore, a vital factor in the protection and promotion of QOL for these older adults [45].

Lastly, social participation is another dimension that most likely acts as a mechanism for improving QOL. Studies have shown that the promotion of social participation among older individuals is a key factor in successful aging [46,47]. Social participation itself, which involves getting up and interacting with others, is a key characteristic of healthy aging [48]. However, as older adults advance in age, social participation changes. These changes could be related to the deteriorating physical and cognitive health capacities of an individual [46]. The key now is to create and/or maintain a healthy active social lifestyle. Therefore, social participation can be recognized as a vital approach for nurturing QOL.

**Figure 1:**
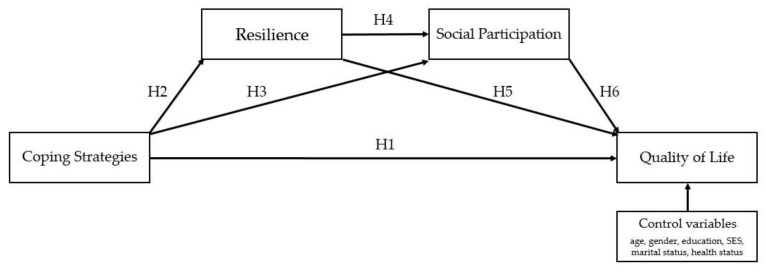
The theoretical framework of the study.

With that being said, the primary objective of the current study is to understand the relationships between coping strategies, resilience, social participation, and QOL among older adults in the Western Philippines. More specifically, the study investigates the parallel and serial mediating role of resilience and social participation within the relationship between coping strategies and QOL, while controlling for the participants’ background demographics (age, gender, educational attainment, SES or average monthly income, marital and health status; see Figure 1 for more details). The general hypotheses are indicated by A to D, and the specific hypotheses are indicated by H1 to H6; A and H1 relate to the same hypothesis. 

In terms of parallel mediation, the following hypotheses were made:

1. Coping strategies are directly associated with QOL (H1).
2. In terms of indirect effects through resilience, higher levels of coping strategies are associated with higher levels of resilience (H2), which in turn are associated with higher levels of QOL (H5).
3. Similarly, indirect effects through social participation indicate that higher levels of coping strategies are associated with higher levels of social participation (H3), which in turn are associated with higher levels of QOL (H6).
  In terms of serial mediation, it has been hypothesized that coping strategies, resilience, social participation, and QOL affect each other sequentially. A more specific hypothesis is provided:
4. High levels of coping strategies lead to high levels of resilience, which in turn lead to high levels of social participation, and ultimately lead to high levels of QOL (path analysis from H2, H4, to H6).

## 2. Method and Materials

### 2.1. Participants and Procedure

The current study was accomplished in the Western Philippines, wherein participants are volunteer older adults residing within the six major towns in the Visayas region, namely: Antique, Aklan, Bacolod, Guimaras, Iloilo, and Roxas. The total population of the region is around 7.5 million, with 4.6% accounting for older adults 60 years old and above. A volunteer sampling method was used, wherein participants were self-selected to become part of the study [49]. Recruitment of the participants was assisted by the Federation of Seniors Citizens Association of the Philippines (FSCAP). Permissions were secured from the respective presidents of each of the FSCAP chapters within the six major towns. Then, permissions from the respective barangay leaders—the basic unit of administration in the Philippines—for a neighborhood typically encompassing from 50 to 100 families, were also obtained. 

Data collection was achieved during the regular assembly of the FSCAP by the primary researcher with the assistance of three nurses, who assisted the participants, especially those who needed help with writing. Additional participants were also selected upon the recommendations of the barangay leaders. Every participating older adult was oriented to the purpose of the study. The research objective was explained carefully, and informed consent was obtained from all of the participants. Emphasis on the privacy and confidentiality of the participants as well as their rights and benefits was also observed. Anonymity was maintained in all the data gathered. In addition, participants are free to withdraw from the study at any time they wish without any consequences. The protocol of the study was reviewed and approved by the panel of evaluators of the University of St. La Salle Graduate Program.

The Sampsize program [50] was used to calculate the minimum sample size. Since there are approximately 472,000 senior adults aged 65 years old and above living in the region, a minimum sample size of 384 participants was needed for this study (with a 5% margin of error and 95% confidence level). Data from 392 older adults were collected. The data show that the majority of the participants are female, with 302 or 77%, while the rest are male, with 90 or 23%. The overall mean age of the participants is 69 years old (the same for males and females).

For the background demographics of the participants, Table 1 shows that most of the participants are college graduates (41%) and are either married (51%) or widowed (35%). The average monthly income is computed between PHP 5000 (approximately around USD 102 using the exchange rate of 1 USD = 49 PHP) and PHP 15,000 (or USD 306). The data show that almost one-third (29%) belong to a high SES status with an average monthly income of above PHP 15,000 (or more than USD 306). As for the participant’s health status, approximately two out of every five (43%) have been diagnosed with a chronic disease, while around 31% reported no chronic disease.

### 2.2. Research Tools

In addition to the relevant background demographics such as age, gender, educational attainment, SES or average monthly income, and marital and health status, several validated instruments were also utilized to collect the participants’ coping strategies, resilience, social participation, and QOL. For the accuracy of data collection, the instruments were translated into the three major local dialects in Western Philippines (Hiligaynon, Kinaraya, and Akeanon). The survey items were then translated back into English to ensure the essence of the survey items was preserved [51].

#### 2.2.1. Coping Strategies

Coping strategies were measured by using the Inventory of Coping Strategies used by the Elderly (ICSUE) developed originally by Robichaud and Lamarre [52] and later adapted in Demers et al.’s [29] study. The 30-item ICSUE is composed of two major components, namely behavioral and cognitive coping strategies [29]. The items are attitudes developed through life experiences that help minimize the functional impacts of an impairment or disability, while at the same time preventing the loss of autonomy. Cronbach [53] alpha reliability of the ICSUE was computed at 0.82, signifying moderate internal consistencies [54]. Sample items include: *I try to see the positive side to aging*, *I keep myself entertained and take advantage of life’s pleasures*, *I ask somebody to remind me to take my medication*, and *I ask somebody to help me move around*. Data were collected using a 4-point Likert [55]-type scale with ratings ranging from 0—not applicable to 3—yes (or often). Computation of the ICSUE was accomplished by determining the frequency of use for each of the strategies.

#### 2.2.2. Resilience Scale

To understand the participants’ level of resilience, the current study used the 14-item Resilience Scale (RS14) developed by Wagnild [56]. The scale captures the various characteristics of resilience, such as equanimity, perseverance, self-reliance, purpose, and existential aloneness or authenticity [56,57,58,59]. Cronbach alpha reliability of the RS14 was computed at 0.91, signifying high internal consistencies. Sample items include: *I usually manage one way or another*, *I can usually find something to laugh about*, and *when I’m in a difficult situation, I can usually find my way out of it*. Data were collected using a 7-point Likert-type scale with ratings ranging from 1—strongly disagree to 7—strongly agree. The RS14 was determined by summing all the items, while the interpretation of the results is as follows: 91 to 98—high resilience, 82 to 90—moderately high resilience, 74 to 81—moderate resilience, 65 to 73—on the low-end resilience, 57 to 64—low resilience, and 14 to 56—very low resilience. 

#### 2.2.3. Social Participation

Social participation was measured with a scale developed by Kubach [60]. The 12-item Social Participation Scale (SP12) consists of concepts of participation that are used to describe an individual’s involvement in a life situation, particularly activities and roles that are valued by the individual as well as by the social environment [60]. Cronbach alpha reliability of the SP12 was computed at 0.89, signifying high internal consistencies. Sample items include: *having interesting recreational or cultural activities to attend*, *communicating/visiting with friends and or family*, and *used a recreation center in your community*. Data were collected using a 4-point Likert-type scale with ratings ranging from 1—very low to 4—very high. The SP12 was calculated by adding up all the items, while the interpretation of the results is as follows: 37 to 48—excellent social participation, 25 to 36—good social participation, 13 to 24—fair social participation, and 1 to 12—poor social participation. 

#### 2.2.4. Quality of Life

The QOL of older adults was measured using the Older People’s Quality of Life Questionnaire (OPQOL) developed by Bowling [61]. The 35-item OPQOL is composed of various issues that represent the following: overall life, health, social relationships and participation, independence, control over life or freedom, home and neighborhood, psychological and emotional well-being, financial circumstances, and religion and culture. To avoid redundancy of measures, the 5 items corresponding to the social relationships and participation were not included in the final analysis of QOL. Cronbach alpha reliability of the OPQOL was computed at 0.70, signifying adequate internal consistencies. Sample items include: *I am healthy enough to get out and about*, *I feel safe where I live, I feel lucky compared to most people*, and *I try to stay involved with things*. Data was collected using a 5-points Likert-type scale with ratings ranging from 1—strongly disagree to 5—strongly agree. Computation of the OPQOL was accomplished by taking the sum of all the items, while interpretation of the results is as follows: 117 to 175—higher QOL, 59 to 116—moderate QOL, and 35 to 58—lower QOL. 

### 2.3. Data Analysis

After all the survey forms were collected, data were encoded and analyzed with the use of the Statistical Package for the Social Sciences (SPSS) software version 20 (IBM Corp., Armonk, NY, USA). Analyses including the descriptive statistics, inter-correlations between the study variables, and reliabilities were computed using SPSS. Structural equation modeling was used to determine the direct and indirect effects (or mediating roles) of the predictor variables (including the bootstrapping of confidence levels and their *p*-values). Structural equation modeling was accomplished with the use of AMOS software version 26 (IBM Corp., Armonk, NY, USA) leased from Hearne software. The bootstrap method (sampling repeated 2000 times) was used to estimate the 95% confidence intervals. As a rule of thumb, confidence intervals should not include zero (0) to denote significant indirect effects [62]. For the model fit, several criteria were used: standardized root means square residual (SRMR; values < 0.08 indicating a good fit); significant Chi-square; Chi-square divided by degrees of freedom (CMIN/df; the ratio should fall between 2 and 5 indicating a reasonable fit); root mean square error of approximation (RMSEA; values < 0.08 indicating a good fit), including 90% confidence interval (90% CI); and goodness of fit index (GFI), and comparative fit index (CFI); both should have values > 0.90 to indicate a good fit [63,64]. 

## 3. Results

Table 2 shows the descriptive statistics, inter-correlations, and reliabilities of the different variables. Findings show that QOL is positively correlated with resilience (*r* = 0.31, *p* < 0.01), coping strategies (*r* = 0.18, *p* < 0.01), and social participation (*r* = 0.45, *p* < 0.01). In addition, social participation is also positively correlated with resilience (*r* = 0.28, *p* < 0.01) and coping strategies (*r* = 0.17, *p* < 0.01). Lastly, coping strategies are also correlated with the participants’ resilience (*r* = 0.25, *p* < 0.01). In essence, resilience, coping strategies, social participation, and QOL of select older adults in the Western Philippines are positively correlated with each other.

For the background demographics, age was found to be positively correlated with coping strategies (*r* = 0.01, *p* < 0.05), denoting that older individuals tend to have higher coping tendencies. Furthermore, age was found to be negatively correlated with resilience (*r* = −0.10, *p* < 0.05), signifying that younger individuals tend to be more resilient.

**Figure 2:**
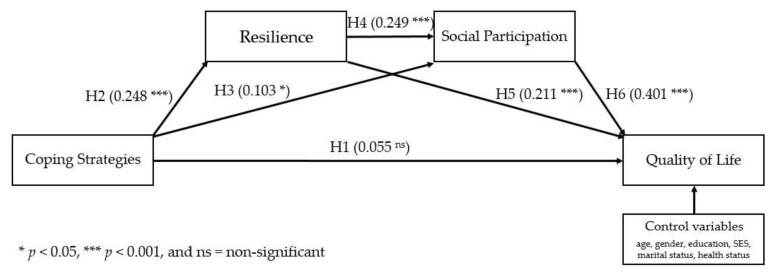
Path analytical model of the inter-relationship between the study variables.

Figure 2 shows the path analytical model tested and the associated standardized regression weights using structural equation modeling. In the figure, coping strategies are regarded as the predictor variable, while QOL is regarded as the outcome variable. In addition, it is also conceptualized that resilience and social participation act as both parallel and serial mediators. Structural equation modelling results exhibited a mediocre model fit with SRMR = 0.05, CMIN (10) = 39.58 with *p* < 0.001, CMIN/df = 3.96, RMSEA = 0.08 (90% CI 0.60 and 0.11), GFI = 0.98, and CFI = 0.89. Table 3 and Table 4 show the direct and indirect effects of the predictor and mediators.

Table 3 indicates that upon controlling for the older adults’ age, gender, educational attainment, SES, and marital and health status, each of the direct effects were all significant (except coping strategies -> QOL or hypothesis A or H1; not supported). Coping strategies were found to have significant direct positive effects on resilience (H2) with β = 0.248, *p* < 0.001 and social participation (H3) with β = 0.103, *p* = 0. 038. These indicate that higher levels of coping strategies are associated with higher levels of resilience and social participation. In addition, resilience was also found to have significant direct positive effects on QOL (H5) with β = 0.211, *p* < 0.001 and social participation (H4) with β = 0.249, *p* < 0.001. These findings denote that higher levels of resilience are associated with higher levels of QOL and social participation. Lastly, social participation was also found to have significant direct positive effects on QOL (H6) with β = 0.401, *p* < 0.001, signifying that higher levels of social participation are associated with higher levels of QOL. 

Table 4 shows the total indirect effects for the parallel and serial mediations (path analysis). Results show that all of the indirect effects are significant, thus supporting hypotheses B, C, and D. More importantly, the parallel mediation paths coping strategies -> resilience -> QOL with total effects of β = 0.052, *p* < 0.001 and coping strategies -> social participation -> QOL with total effects of β = 0.041, *p* = 0.046, both denoted full mediation. In other words, the direct effect of coping strategies -> QOL is not significant. Therefore, the mediators resilience and social participation both fully mediate the relationship between coping strategies and QOL. Furthermore, the overall serial mediation (serial path analysis from coping strategies -> resilience -> social participation -> QOL) is also significant with total effects of β = 0.062, *p* < 0.001, denoting that high levels of coping strategies lead to high levels of resilience, which in turn lead to high levels of social participation, and ultimately lead to high levels of QOL.

## 4. Discussions

The current study investigated the parallel and serial mediating role of resilience and social participation within the relationship between coping strategies and QOL while controlling for the participants’ background demographics: age, gender, educational attainment, SES, and marital and health status. Controlling for the participants’ background demographics was conducted deliberately to do away with their effects on the relationship between coping strategies and QOL [65,66]. Several distinct findings are noted. First, when taken alone, coping strategies are not associated with QOL. Importantly, this result does not contradict previous research, which found that coping strategies improve QOL. However, it rather emphasized that coping strategies by themselves are not sufficient to improve QOL.

Second, findings confirmed the mediating role of resilience and social participation within the relationship of coping strategies and QOL. As previously noted, coping strategies alone are insufficient to improve QOL. The only way to improve QOL is through resiliency, as well as the ability to participate in social activities. Lastly, the findings also confirmed the sequential nature of the relationships between coping strategies, resilience, social participation, and QOL. The findings consistently showed resilience as one of the major contributors to successful aging [67], while also confirming the role social participation plays in improving the mental health of older adults [68], and subsequently improving their QOL [69].

The literature shows that resilience is a developmental and dynamic process that originates from childhood and continues until the end of life [70]. By nature, individuals are able protect themselves, adapt, and persevere [71]. More specifically, resilience is a measure of how well an individual can overcome barriers and function effectively despite adverse circumstances [35], while protective factors, such as coping strategies and social participation, when utilized by the individual, can also help the person overcome adverse events. Hence, when confronted with an uneventful situation, one must know how to resolve and address the problem, which will then control the outcome. Overcoming adversity and growing afterward resulting from the said experience is true resilience [72].

In application, this can be seen within an individual’s ability to manage the aging process and maintain a sense of purpose and vitality. It is a dynamic distinctive characteristic that may change according to the contextual situation. Consequently, coping strategies, resilience, and social participation can be considered cognitive and behavioral tools that can help older adults cope with life changes resulting from aging [73]. Furthermore, according to the theory of activity for successful aging, to preserve old roles, one must have new roles to be discovered or new things must be innovated [74]. Therefore, social procedures and alterations of social goals are necessary for the establishment of a dynamic life, which can be achieved through social support and social engagement among older adults. In essence, social participation is one of the effective elements that help facilitate later QOL. For the current group of participants, it can be assumed that as the majority of the participants are within the 60–69 age group, they might have more opportunities to participate in community activities. After retirement, most of them probably still have better health conditions, making it possible to live an active social life, such as by joining organizations among senior citizens.

It would seem that the results of the current study were supported by previous findings. Most older adults demonstrated high well-being and better QOL despite their age. Moreover, older adults with a positive outlook in life have been associated with high resilience and successful aging. Research suggests that older adults can obtain an improved QOL with high resilience, which includes happiness, better health status, and contentment with life. Therefore, they are more likely to age successfully. In addition, the findings indicated that although coping strategies are important, both resilience and social participation are also key predictors of positive and successful aging. Social participation among older adults in the Philippines might include activities of daily living, social connections, spirituality, and community life. Maintaining an active and engaging life is, therefore, important. Enhancing the social participation of older adults is a vital factor in successful aging, which translates into high QOL [75].

### Limitations of the Study

Since the current study was conducted in 2019, the findings will be limited to the existing situations and conditions of the older adults living in the Western Philippines before the COVID-19 pandemic. In addition, since the participants are mostly volunteering older adult members of FSCAP, it might imply that most of them are already living an active retirement. Hence, the findings will also be limited to older adults who have a similar lifestyle. 

## 5. Conclusions

Considering the various concepts and issues mentioned, it is high time that attention is focused on areas relevant to the well-being of the older adult population. Within the Western Philippines, little is known regarding the diverse life experiences of older adults and how they cope with challenges. Findings such as the importance of social participation, as noted within the study, have a very positive effect in terms of enhancing the QOL of older adults. In today’s society, social isolation is a greater risk for older people, presumably due to the various limitations within the dimensions of life of an older adult that compromise their ability to maintain social connections. In the absence of social participation among older adults, the risk of depressive disorders becomes greater, leading eventually to a greater rate of morbidity and mortality. Thus, social engagement among older adults is of utmost importance as it provides meaning and contributes to their subjective well-being and overall QOL.

In future studies, researchers could collaborate with local authorities in organizing workshops that simultaneously foster social interactions and increase older adults’ self-worth. In addition, qualitative studies or narratives that describe how older adults interact with their children are also a topic of interest. The perspectives on how older adults can promote their QOL while living with their children can be seen as a uniquely Asian viewpoint.

## Figures and Tables

**Table 1:** Background demographics of the participants.

Category	Groups	*n*	%
Educational Attainment	Elementary	93	25
	High School	114	29
	College	159	41
	Graduate	26	7
Marital Status	Single	45	12
	Married	199	51
	Widow	136	35
	Separated	12	3
Average Monthly Income ^1^	Low (below 5000)	92	24
	Middle (5000 to 15,000)	187	48
	High (above 15,000)	113	29
Health Status	0 Chronic Disease	120	31
	1 Chronic Disease	167	43
	2 Chronic Diseases	44	11
	3 Chronic Diseases	43	11
	4 Chronic Diseases	18	5

Notes. Mean age of participants = 69 years old. ^1^ Monthly average income or SES is in Philippine Peso (PHP); 1 USD = 49 PHP. N = 392.

**Table 2:** Descriptive statistics, inter-correlations, and reliabilities for the study variables.

Variables	1	2	3	4	5	6	7
1. Quality of life	1						
2. Coping strategies	0.18 **	1					
3. Resilience	0.31 **	0.25 **	1				
4. Social participation	0.45 **	0.17 **	0.28 **	1			
5. Age	ns	0.01 *	−0.10 *	ns	1		
6. SES	ns	ns	ns	ns	ns	1	
7. Health status	ns	ns	ns	ns	ns	ns	1
Minimum value	71	26	57	15	60	3000	0
Maximum value	137	92	98	48	96	500,000	4
Mean	112.51	60.33	82.55	35.68	69.27	16,307.33	1.16
SD	9.03	9.56	11.77	6.52	6.50	34,371.66	1.12
Alpha reliability	0.70	0.82	0.91	0.89			

Notes. N = 392, SES = socioeconomic status, ns = non-significant, and SD = standard deviation. ** *p* < 0.01, * *p* < 0.05. Numbers 1 to 7 correspond to the variables.

**Table 3:** Direct effects of the predictor variables and mediators.

Direct Effects	B	SE	β	95% CI	*p*
A or H1: Coping strategies → Quality of life	0.053	0.043	0.055	[−0.02, 0.13]	0.219
H2: Coping strategies → Resilience	0.305	0.060	0.248	[0.16, 0.34]	<0.001
H3: Coping strategies → Social participation	0.071	0.034	0.103	[0.02, 0.19]	0.038
H5: Resilience → Quality of life	0.164	0.035	0.211	[0.13, 0.29]	<0.001
H4: Resilience → Social participation	0.138	0.028	0.249	[0.16, 0.33]	<0.001
H6: Social participation → Quality of life	0.564	0.063	0.401	[0.32, 0.48]	<0.001

Notes. B = Regression coefficient, SE = standard error, β = standardized coefficient, and CI = confidence interval.

**Table 4:** Indirect effects of the predictor variables and mediators.

Indirect Effects	B	SE	β	95% CI	*p*
Parallel mediation					
Coping strategies → Resilience → Social participation ^1^	0.042	0.013	0.062	[0.02, 0.07]	0.001
B: Coping strategies → Resilience → Quality of life ^2^	0.050	0.028	0.052	[0.03, 0.08]	<0.001
C: Coping strategies → Social participation → Quality of life ^2^	0.040	0.019	0.041	[0.01, 0.08]	0.046
Resilience → Social participation → Quality of life ^1^	0.078	0.055	0.100	[0.05, 0.11]	0.001
Serial mediation					
D: Coping strategies → Resilience → Social participation → Quality of life	0.024	0.023	0.062	[0.01, 0.04]	<0.001

Notes. B = Regression coefficient, SE = standard error, β = standardized coefficient, and CI = confidence interval. ^1^ Partial mediation and ^2^ full mediation.

## Data Availability

Data for the current study is available at https://doi.org/10.6084/m9.figshare.14399243.v1 (accessed on 2 April 2021).

## References

[1] Wang S. (2020). Spatial patterns and social-economic influential factors of population aging: A global assessment from 1990 to 2010. Soc. Sci. Med..

[2] Philippine Statistics Authority (2015). Facts on Senior Citizen.

[3] Philippine Statistics Authority (2019). Women and men in Western Visayas: 2019 Statistical Hanbook.

[4] Roxas J.N.R., Fillone A.M. (2017). Co-benefit analysis of the proposed Panay-Guimaras-Negros Bridge Project, Western Visayas, Philippines. Transp. Res. Procedia.

[5] Philippine Statistics Authority Gross Domestic Product of the Philippines Highlights for 2018. https://psa.gov.ph/grdp/highlights-id/138509.

[6] Ranola A. Combating Elderly Poverty in the Philippines. https://borgenproject.org/elderly-poverty-in-the-philippines/.

[7] Momblan G.W. Visayas Senior Citizens Start Receiving Social Pensions. https://www.pna.gov.ph/articles/1101297.

[8] Reyes C.M., Arboneda A.A., Asis R.D. (2019). Silver Linings for the Elderly in the Philippines: Policies and Programs for Senior Citizens.

[9] Badana M.A.N.S., Andel R. (2018). Aging in the Philippines. Gerontologist.

[10] Carandang R.R., Asis E., Shibanuma A., Kiriya J., Murayama H., Jimba M. (2019). Unmet Needs and Coping Mechanisms Among Community-Dwelling Senior Citizens in the Philippines: A Qualitative Study. Int. J. Environ. Res. Public Health.

[11] Esteban R.C. (2015). Thinking about Aging: Experience, Identity and Meaning among an Elderly Population in the Philippines. Adv. Aging Res..

[12] Faner M.R., Chiong M.A.D. (2020). Clinical Profiles and Outcomes of the Most Common Inherited Metabolic Diseases in the Philippines: A Review of the National Institutes of Health—Institute of Human Genetics Metabolic Registry. Acta Med. Philipp..

[13] Kerschner H., Pegues J.A.M. (1998). Productive Aging: A Quality of Life Agenda. J. Am. Diet. Assoc..

[14] World Health Organization (1947). The Constitution of the World Health Organization.

[15] Spitzer W.O. (1987). State of science 1986: Quality of life and functional status as target variables for research. J. Chronic Dis..

[16] Medvedev O.N., Landhuis C.E. (2018). Exploring constructs of well-being, happiness and quality of life. PeerJ.

[17] Aldwin C.M., Yancura L.A., Boeninger D.K., Aldwin C.M., Park C.L., Spiro A., III (2007). Coping, health, and aging. Handbook of Health Psychology and Aging.

[18] Borowiak E., Kostka T. (2004). Predictors of quality of life in older people living at home and in institutions. Aging Clin. Exp. Res..

[19] Harms C.A., Cohen L., Pooley J.A., Chambers S.K., A Galvao D., Newton R.U. (2018). Quality of life and psychological distress in cancer survivors: The role of psycho-social resources for resilience. Psycho-Oncology.

[20] Mayordomo T., Viguer P., Sales A., Satorres E., Melendez J.C. (2016). Resilience and Coping as Predictors of Well-Being in Adults. J. Psychol..

[21] Galiana L., Gutiérrez M., Sancho P., Francisco E.-H., Tomas J.M. (2016). Socio-Demographic Variables and Successful Aging of the Angolan Elderly. Scientifica.

[22] Wieder-Huszla S., Szkup M., Jurczak A., Samochowiec A., Samochowiec J., Stanisławska M., Rotter I., Karakiewicz B., Grochans E. (2014). Effects of Socio-Demographic, Personality and Medical Factors on Quality of Life of Postmenopausal Women. Int. J. Environ. Res. Public Health.

[23] Hajian-Tilaki K., Heidari B., Hajian-Tilaki A. (2017). Are Gender Differences in Health-related Quality of Life Attributable to Sociodemographic Characteristics and Chronic Disease Conditions in Elderly People?. Int. J. Prev. Med..

[24] Galiana L., Tomás J.M., Fernández I., Oliver A. (2020). Predicting Well-Being Among the Elderly: The Role of Coping Strategies. Front. Psychol..

[25] Hunt S., Wisocki P., Yanko J. (2002). Worry and use of coping strategies among older and younger adults. J. Anxiety Disord..

[26] Peláez-Ballestas I., Boonen A., Vázquez-Mellado J., Reyes-Lagunes I., Hernández-Garduño A., Goycochea M.V., Bernard-Medina A.G., Rodríguez-Amado J., Casasola-Vargas J., Garza-Elizondo M.A. (2015). Coping Strategies for Health and Daily-Life Stressors in Patients With Rheumatoid Arthritis, Ankylosing Spondylitis, and Gout. Medicine.

[27] Greenglass E.R., Frydenberg E. (2002). Proactive coping and quality of life management. Beyond Coping: Meeting Goals, Visions, and Challenges.

[28] Chabowski M., Jankowska-Polanska B., Lomper K., Janczak D. (2018). The effect of coping strategy on quality of life in patients with NSCLC. Cancer Manag. Res..

[29] Demers L., Robichaud L., Noreau L., Gélinas I., Desrosiers J. (2008). Coping Strategies and Social Participation in Older Adults. Gerontology.

[30] Atal S., Cheng C. (2016). Socioeconomic health disparities revisited: Coping flexibility enhances health-related quality of life for individuals low in socioeconomic status. Health Qual. Life Outcomes.

[31] Gattino S., Rollero C., De Piccoli N. (2014). The Influence of Coping Strategies on Quality of Life from a Gender Perspective. Appl. Res. Qual. Life.

[32] Kupcewicz E., Grochans E., Kadučáková H., Mikla M., Jóźwik M. (2020). Analysis of the Relationship between Stress Intensity and Coping Strategy and the Quality of Life of Nursing Students in Poland, Spain and Slovakia. Int. J. Environ. Res. Public Health.

[33] Dockendorff D.C.T. (2013). Healthy Ways of Coping With Losses Related to the Aging Process. Educ. Gerontol..

[34] Philippine Statistics Authority (2020). Special Release: Poverty Statistics Among Families in Western Visayas.

[35] Southwick S.M., Bonanno G.A., Masten A., Panter-Brick C., Yehuda R. (2014). Resilience definitions, theory, and challenges: Interdisciplinary perspectives. Eur. J. Psychotraumatol..

[36] Kais S.M., Islam S. (2016). Community Capitals as Community Resilience to Climate Change: Conceptual Connections. Int. J. Environ. Res. Public Health.

[37] Windle G. (2010). What is resilience? A review and concept analysis. Rev. Clin. Gerontol..

[38] Lawford J., Eiser C. (2001). Exploring links between the concepts of Quality of Life and resilience. Pediatr. Rehabil..

[39] Lu C., Yuan L., Lin W., Zhou Y., Pan S. (2017). Depression and resilience mediates the effect of family function on quality of life of the elderly. Arch. Gerontol. Geriatr..

[40] Guccione A.A. (2014). Resilience and Self-efficacy As Mediators of Quality of Life in Geriatric Rehabilitation. Top. Geriatr. Rehabil..

[41] Gerino E., Rollè L., Sechi C., Brustia P. (2017). Loneliness, Resilience, Mental Health, and Quality of Life in Old Age: A Structural Equation Model. Front. Psychol..

[42] Carandang R., Shibanuma A., Asis E., Chavez D., Tuliao M., Jimba M. (2020). “Are Filipinos Aging Well?”: Determinants of Subjective Well-Being among Senior Citizens of the Community-Based ENGAGE Study. Int. J. Environ. Res. Public Health.

[43] Chung M.S. (2011). Resilience, Coping Methods, and Quality of Life in Middle-aged Women. J. Korean Acad. Psychiatr. Ment. Health Nurs..

[44] Yashin A.I., Arbeev K.G., Kulminski A., Akushevich I., Akushevich L., Ukraintseva S.V. (2007). Health decline, aging and mortality: How are they related?. Biogerontology.

[45] MacLeod S., Musich S., Hawkins K., Alsgaard K., Wicker E.R. (2016). The impact of resilience among older adults. Geriatr. Nurs..

[46] Aroogh M.D., Shahboulaghi F.M. (2020). Social participation of older adults: A concept analysis. Int. J. Community Based Nurs. Midwifery.

[47] McKibbin C., Lee A., Steinman B.A., Carrico C., Bourassa K., Slosser A. (2016). Health Status and Social Networks as Predictors of Resilience in Older Adults Residing in Rural and Remote Environments. J. Aging Res..

[48] Choi J., Cho Y., Kim Y., Lee S., Lee J., Yi Y., Tak Y., Hwang H., Lee S., Park E. (2021). The Relationship of Sitting Time and Physical Activity on the Quality of Life in Elderly People. Int. J. Environ. Res. Public Health.

[49] Cohen L., Manion L., Morrison K. (2007). Research Method in Education.

[50] Glaziou P. Sampsize. http://sampsize.sourceforge.net/iface/.

[51] Son J. (2018). Back translation as a documentation tool. Int. J. Transl. Interpreting Res..

[52] Robichaud L., Lamarre C. (2002). Developing an Instrument for Identifying Coping Strategies Used by the Elderly to Remain Autonomous. Am. J. Phys. Med. Rehabil..

[53] Cronbach L.J. (1951). Coefficient alpha and the internal structure of tests. Psychometrika.

[54] Nunnally J.C. (1970). Introduction to Psychological Measurement.

[55] Likert R. (1932). A Technique for the Measurement of Attitudes.

[56] Wagnild G.M. (2011). The Resilience Scale User’s Guide for the US English Version of the Resilience Scale and the 14-Item Resilience Scale (RS-14).

[57] Wagnild G. (2009). A Review of the Resilience Scale. J. Nurs. Meas..

[58] Wagnild G.M., Collins J.A. (2009). Assessing Resilience. J. Psychosoc. Nurs. Ment. Health Serv..

[59] Wagnild G.M., Young H.M. (1993). Development and psychometric evaluation of the Resilience Scale. J. Nurs. Meas..

[60] Kubach O. (2014). Examining Social Participation of Older Adults to Help Create an Age Friendly Community. Master’s Thesis.

[61] Bowling A. (2009). The psychometric properties of the older people’s quality of life questionnaire, compared with the CASP-19 and the WHOQOL-OLD. Curr. Gerontol. Geriatr. Res..

[62] Hayes A.F. (2009). Beyond Baron and Kenny: Statistical Mediation Analysis in the New Millennium. Commun. Monogr..

[63] Hu L., Bentler P.M. (1999). Cutoff criteria for fit indexes in covariance structure analysis: Conventional criteria versus new alternatives. Struct. Equ. Model. A Multidiscip. J..

[64] Byrne B.M. (2010). Structural Equation Modeling with AMOS. Basic Concepts, Applications, and Programming.

[65] Spector P.E., Brannick M.T. (2010). Methodological Urban Legends: The Misuse of Statistical Control Variables. Organ. Res. Methods.

[66] Frölich M. (2008). Parametric and Nonparametric Regression in the Presence of Endogenous Control Variables. Int. Stat. Rev..

[67] Hildon M.Z., Montgomery S., Blane M.D., Wiggins R.D., Netuveli G. (2009). Examining Resilience of Quality of Life in the Face of Health-Related and Psychosocial Adversity at Older Ages: What is “Right” About the Way We Age?. Gerontologist.

[68] Takagi D., Kondo K., Kawachi I. (2013). Social participation and mental health: Moderating effects of gender, social role and rurality. BMC Public Health.

[69] Tobin M.C., Drager K.D., Richardson L.F. (2014). A systematic review of social participation for adults with autism spectrum disorders: Support, social functioning, and quality of life. Res. Autism Spectr. Disord..

[70] Masten A.S., Barnes A.J. (2018). Resilience in Children: Developmental Perspectives. Children.

[71] Reivich K., Shatté A. (2002). The Resilience Factor: 7 Essential Skills for Overcoming Life’s Inevitable Obstacles.

[72] Yates T., Tyrell F., Masten A. (2015). Resilience Theory and the Practice of Positive Psychology From Individuals to Societies. Posit. Psychol. Pract. Promot. Hum. Flourishing Work. Health Educ. Everyday Life.

[73] León-Navarrete M.M., Flores-Villavicencio M.E., Mendoza-Ruvalcaba N., Colunga-Rodríguez C., Salazar-Garza M.L., Sarabia-López L.E., Albán-Pérez G.G. (2017). Coping Strategies and Quality of Life in Elderly Population. Open J. Soc. Sci..

[74] Katz S. (2000). Busy Bodies: Activity, aging, and the management of everyday life. J. Aging Stud..

[75] Douglas H., Georgiou A., Westbrook J. (2017). Social participation as an indicator of successful aging: An overview of concepts and their associations with health. Aust. Health Rev..

